# Follow-up assessment of visceral leishmaniasis treated patients and the impact of COVID-19 on control services in Nepal

**DOI:** 10.1186/s41182-023-00549-1

**Published:** 2023-10-20

**Authors:** Anand Ballabh Joshi, Megha Raj Banjara, Murari Lal Das, Nav Raj Bist, Krishna Raj Pant, Uttam Raj Pyakurel, Gokarna Dahal, Krishna Prasad Paudel, Chuman Lal Das, Axel Kroeger, Abraham Aseffa

**Affiliations:** 1 Public Health and Infectious Disease Research Center (PHIDReC), Kathmandu, Nepal; 2 Central Department of Microbiology, Tribhuvan University, Kirtipur, Kathmandu, 44601 Nepal; 3 UNICEF/UNDP/World Bank/WHO Special Programme for Research and Training in Tropical Diseases (TDR), Geneva, Switzerland; 4 Epidemiology and Disease Control Division, Department of Health Services, Teku, Kathmandu, Nepal; 5 Centre for Medicine and Society/Institute for Infection Prevention, Albert-Ludwigs-University, Freiburg, Germany

**Keywords:** Visceral leishmaniasis, Treatment follow-up, Relapse, Impact of COVID-19, Public health programmes

## Abstract

**Background:**

Follow-up assessment of visceral leishmaniasis (VL) treated cases is important to monitor the long term effectiveness of treatment regimens. The main objective of this study was to identify the gaps and challenges in the follow-up of treated VL cases, to monitor treatment outcome and to assess the impact of COVID-19 on VL elimination services and activities.

**Methods:**

Clinicians treating VL patients, district focal persons for VL, and patients treated for VL in seven high endemic districts in Nepal during 2019–2022 were interviewed to collect data on challenges in the follow-up of VL treated patients as per national strategy.

**Results:**

Follow up status was poor in two districts with the largest number of reported cases. The majority of cases were children under 10 years of age (44.2%). Among 104 VL treated cases interviewed, 60.6% mentioned that clinicians had called them for follow-up but only 37.5% had complied. Among 112 VL treated cases followed up, 8 (7.14%) had relapse and 2 (1.8%) had PKDL. Among 66 cases who had VL during the COVID-19 lock down period, 32 (48.5%) were diagnosed within 1 week; however, 10 (15.1%) were diagnosed only after 4 weeks or more. During the COVID-19 pandemic, there was no active search for VL because of budget constraints and lack of diagnostic tests, and no insecticide spraying was done.

**Conclusion:**

Relapses and PKDL are challenges for VL elimination and a matter of concern. Successful implementation of the national strategy for follow up of treated VL cases requires addressing elements related to patients (awareness, transport, communication) clinicians (compliance) and organization of service delivery (local health worker training and deployment). COVID-19 did not have much impact on VL diagnosis and treatment; however, public health programmes including active case detection and insecticide spraying for vector control were severely reduced.

## Introduction

Bangladesh, India and Nepal constituted > 50% of the global visceral leishmaniasis (VL) burden [1]. These countries signed a Memorandum of Understanding (MoU) in 2005 to eliminate VL in the Indian sub-continent by 2015; the elimination target was extended to 2020 [2] and none of the countries has verified the VL elimination to date. The target of the Regional VL Elimination Programme is VL incidence below 1 per 10,000 population at district level in Nepal [3]. The WHO NTD Roadmap 2021–2030 has defined VL elimination as < 1% case fatality rate due to primary visceral leishmaniasis. It has targeted 32 countries to be validated for elimination by 2023, 56 by 2025 and 64 by 2030.

Active case detection, improved vector control and increased awareness of health staff have contributed to a substantial reduction of VL incidence. Treatment with miltefosine and single-dose liposomal amphotericin B (LAmB) has contributed to improvements in VL case management [4]. The countries of the Indian sub-continent have adopted single dose LAmB as the first option for VL treatment, and miltefosine plus paromomycin as second line drugs to replace miltefosine monotherapy in the VL elimination initiative since 2014. Effective treatment is key to improving patient outcomes and reducing disease transmission. Successful VL treatment improves the general condition of the patient, resolves fever (in most cases by the end of the week), and causes regression of splenomegaly. A good indicator of definitive cure is the absence of clinical relapse at 6 months. Extended follow-up till 36 months in India and 48 months in Bangladesh identified additional relapses, suggesting that sentinel follow-up of at least 12-months is useful as a programmatic tool to better identify and quantify relapses. There is a significant relationship between the treatment regimens for VL and the development of PKDL and relapse [5, 6].

Nepal had around 1 million cases of COVID-19 with 12,000 deaths. Nepal had major peaks of COVID-19 in June–December 2020, April–November 2021, and January–February 2022 [7]. A nationwide lock-down due to COVID-19 came into force in Nepal on 24 March 2020 disrupting essential health services [8]. The impact of these public health and social measures on disease control programmes is not yet well investigated in Nepal. There are indications from other low and middle income countries that already fragile health systems have failed to cope with COVID-19 and mitigate its consequences despite the different strategies and measures taken. Although anecdotal evidence suggests that both health care seeking behaviour and access to care for VL have been negatively affected, the actual impact of the pandemic on VL elimination activities has not been assessed to date.

Studies demonstrated that there was relapse of VL up to 7% and development of PKDL among 3–25% of treated VL cases in Bangladesh and India [5, 6]. Assessment of the health status of treated VL cases including relapse, PKDL, anaemia, wasting and other co-morbid illnesses is essential to monitor the effectiveness of VL treatment regimens. Evidence on health system constraints, gaps and challenges that hinder effective outcome can inform the design of better strategies.

The main objective of this study was to identify the gaps and challenges in the follow-up of treated VL cases, to monitor the treatment outcome and to assess the impact of COVID-19 on VL control services to generate the evidence base for strengthening the national programme in follow-up of treated VL cases.

## Methods

### Ethical approval, consent from the participants and safety issues

Ethical approvals were obtained from World Health Organization Ethical Review Committee (WHO-ERC Regd. No. 0003531) and Ethical Review Board of Nepal Health Research Council (NHRC Ref. No. 3089). Past VL cases were interviewed and examined and blood samples were collected upon written informed consent from each participant. Clinicians and district VL focal persons were also interviewed after getting written informed consent.

The study team members were equipped with COVID-19 protective devices, masks, gloves, face shields and hand sanitizers. The participant was requested to use a mask and hand sanitizer was provided. Physical distance was maintained as possible.

### Study design

This implementation research was conducted to identify the gaps and constraints in follow-up of treated VL patients as per national kala-azar elimination programme guidelines. VL cases in seven VL endemic districts treated within the last 2 years from 2019 to 2021 were followed up both retrospectively and prospectively in 2022 for a year at three time points (initial, second follow up at 6 months of first follow up and third follow up at 12 months of first follow up) to monitor clinical improvement including relapse or other consequences. We also documented the impact of COVID-19 on VL control services and activities.

### Study sites and population

**Fig. 1:**
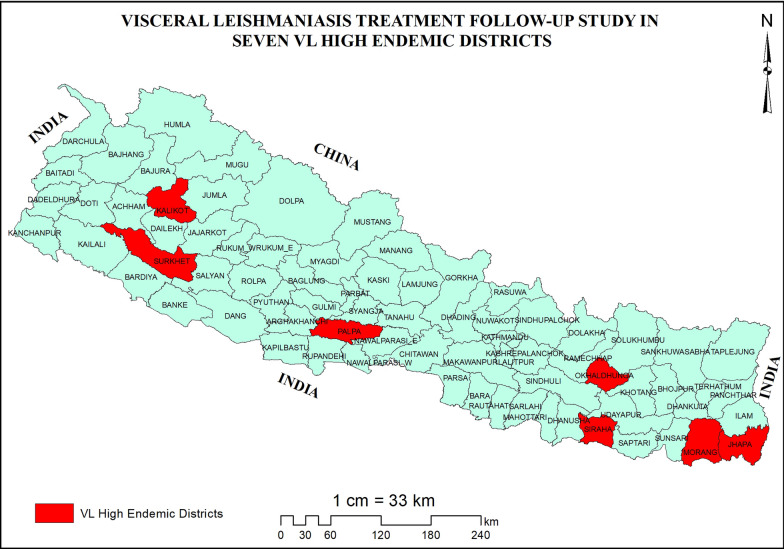
Map showing the location of study areas

Follow-up of treated VL cases was conducted in seven VL high endemic districts Jhapa, Morang, Siraha, Okhaldhunga, Palpa, Surkhet and Kalikot (Fig. 1). The number of VL cases in Nepal during 2019–2022 were 216, 186, 212 and 322 respectively and maximum number of cases were from these selected districts [9]. We included focal persons of VL in district health office, clinicians treating of VL cases, and patients treated for VL during 2019–2022.

Eligible participants, were previously treated VL patients who had completed treatment within the last 2 years, and were resident of the previously mentioned high burden districts identified for the study. They were required to be available for further follow-up, and agree to participate in the study. VL focal persons in the study districts and at national level were also interviewed.

There were three cohorts of treated VL patients that were followed up during this study: (i) cases who had completed treatment more than 1 year but less than 2 years ago were followed up retrospectively, (ii) cases who completed treatment less than 1 year ago were followed up retrospectively and prospectively, and (iii) cases with treatment completion after initiation of the study were followed up prospectively.

### Sample size and sampling

Treated VL cases within the last 2 years from the same districts were followed up for monitoring of outcome. A total of 112 VL treated patients were followed up, 106 were followed up initially, 80 in second follow-up at 6 months of initial follow up and 82 in third follow-up in 1 year of initial follow-up. Sixty six patients who reported to have VL during the pandemic of COVID-19 were interviewed to collect data on barriers and enablers of access to VL care services during the pandemic. All seven focal persons for VL in the districts were interviewed to collect data on the impact of COVID-19.

### Co-ordination with national VL programme

The Epidemiology and Disease Control Division (EDCD) of the Ministry of Health and Population of Nepal collaborated in the study. VL programme staff from the 3 levels (district, provincial and national) participated in the coordination of the research activities of patient follow up.

### Interview of clinicians, focal persons of VL and patients on follow-up and the impact of COVID-19

Data on the status of treated VL patients were collected from VL focal persons in the selected district health offices through interviews using semi-structured questionnaires. Clinicians and health care providers at the district level were interviewed to obtain information on gaps, challenges and opportunities of follow up. Statistical information on follow up was gathered at districts, assessed and compared to previous periods. Frequency and timing of indoor residual spraying and other vector control operations were documented. Challenges faced in supply chain management, in particular of drugs and diagnostics by the programme and barriers to access experienced by patients during the pandemic were explored.

Followed up VL patients were interviewed on barriers of follow-up from their perspectives. It included compliance of follow-up and the reasons for reduced compliance. During the follow-up period, VL patients diagnosed and treated during the COVID-19 pandemic were interviewed on any delay in seeking health care, diagnostic and treatment delays, and on barriers and enabling factors for seeking and receiving VL care.

### Monitoring follow-up of treated VL cases

VL cases who received treatment within the last 2 years were followed up by trained clinicians from government or community hospitals who had experience in VL treatment.

**Fig. 2:**
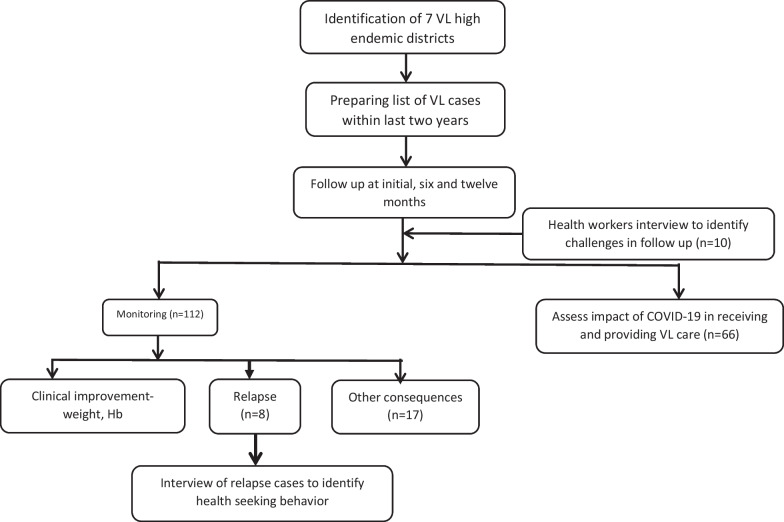
Flow chart of the methods

The key parameters for follow-up included body weight, haemoglobin, spleen size, relapse and PKDL. The detailed address of VL patients who completed treatment within the last 2 years were obtained from health service records and they were followed through house visits upon participant consent. The follow-up visits were conducted at 6 and 12 months of the first enrollment. Therefore, the total follow-up period was from 6 to 36 months in three different cohorts of treated cases. At each of the two follow up visits, medical history was taken, physical examination done and hemoglobin tested for each patient. Confirmatory parasitological tests were done for patients symptomatic with VL to detect relapse cases and PKDL. PKDL was considered probable when a patient with suggestive new skin lesions (hypopigmented macules, papules, nodules or a combination of these) were positive for rK39. Patients with probable PKDL were referred to a VL hospital for confirmation with demonstration of parasites by microscopy (Fig. 2).

### Data management and analysis

Data were entered and analyzed using IBM SPSS version 25. Descriptive analysis was performed. Demographic characteristics of past VL cases, follow-up status and challenges of follow-up were analyzed. Weight and haemoglobin level was measured and compared against earlier values. Gaps and challenges for follow-up were assessed.

## Results

### Gaps and challenges in the follow-up of treated VL patients as per national strategy

We interviewed 10 health workers from six districts (the clinicians involved in patient follow up in Jhapa and Morang were same and in some districts VL focal persons were not available during data collection) including 6 doctors and 4 focal persons of VL on follow-up of treated VL patients. Among them, 3 had more than 10 years of work experience. Most of the health workers mentioned that in their district single dose liposomal amphotericin B was used for the treatment of VL in most cases and multiple dose liposomal amphotericin B and miltefosine were used in others.

Considering the number of VL cases reported in recent years, the follow-up status was poor in Okhaldhunga and Kalikot districts which had reported a large number of VL cases. In other districts, varying numbers of treated VL cases were followed up which was satisfactory according to the reported number of VL cases. Among the 10 health workers interviewed, 4 did follow-up at 12 months after treatment, and 3 at 6 months. For follow-up, the VL treated cases came to the hospital. One health worker did the follow-up through telephone and another one did it through a local health worker. During the follow-up visit, health workers monitored fever, spleen size, weight and haemoglobin. Very few health workers monitored skin lesions. Among 10 health workers, 7 reported that they did not have any problem when following up the VL cases.

As problems in the follow-up, it was mentioned that some patients did not come for follow-up due to lack of awareness or due to distance of long walking hours, particularly for children, and difficult topography in the hilly districts.

It was also mentioned that follow-up could be facilitated by training local health workers to conduct it and through telephone communication.

### Investigation of relapse in treated VL cases and their health seeking behavior

We interviewed 104 patients treated for VL out of 112 cases who were clinically followed up. Among them, the majority were from Okhaldhunga (37, 35.6%) and Kalikot (35, 33.6%) districts, which are both hilly districts. The majority of VL treated cases were children less than 10 years of age (46, 44.2%). Of the 104 cases, 55 (52.9%) were male and 49 (47.1%) were female, the majority had only primary level education (37, 35.6%) or were illiterate (32, 30.7%). The VL cases were mostly school students, small children, labourers or farmers (Table 1).

**Table 1:** Demographic characteristics of the past VL cases on treatment follow-up

Characteristics	Numbers
Past VL cases interviewed in the districts	n = 104
Jhapa	3 (2.9)
Morang	6 (5.7)
Siraha	6 (5.7)
Surkhet	8 (7.7)
Palpa	9 (8.6)
Okhaldhunga	37 (35.6)
Kalikot	35 (33.6)
Age (years)	n = 104
< 10	46 (44.2)
10–20	13 (12.5)
20–30	8 (7.7)
30–40	14 (13.4)
40–50	10 (9.6)
> 50	13 (12.5)
Gender	n = 104
Male	55 (52.9)
Female	49 (47.1)
Education level	n = 104
Primary level	37 (35.6)
Secondary level	17 (16.3)
Higher level	3 (2.9)
Informal education	15 (14.4)
Illiterate	32 (30.7)
Occupation	n = 104
Farmer	16 (15.4)
House wife	9 (8.6)
Business	3 (2.9)
Student	31 (29.8)
Labour	15 (14.4)
Unemployment	1 (0.9)
Office job	2 (1.9)
Others (small children)	27 (25.9)

Among the 104 cases, 102 (98%) had received treatment in a government hospital. Most of the cases (77, 74%) received single dose liposomal amphotericin B (LAmB, 10 mg/Kg body weight) but 15 (14.4%) received multiple doses of LAmB (5mg/Kg body weight a day for 3 days). Six (5.7%) received only miltefosine (100 mg per day for 28 days) and 3 (2.8%) received LAmB (5 mg/Kg single dose) plus miltefosine (2.5 mg/Kg/per day for 7 days). Among 104 VL treated cases, 63 (60.6%) mentioned that the physician called them for follow-up, whereas 41 (39.4%) had no such call. Only 39 (37.5%) of those invited VL treated cases actually went for follow-up. During follow-up, the physician asked about their health status (35, 89.7%), monitored fever (28, 26.9%), measured the spleen size (25, 64.1%), measured the weight (15, 38.4%), monitored the haemoglobin (22, 56.4%) and observed the skin lesions (3, 7.7%). Only 17 (16.3%) cases reported that they had suffered from some health problems (Table 2).

**Table 2:** Interview of past VL cases on treatment follow-up

Characteristics	Numbers
Place of VL diagnosis	n = 104
Private hospital	2 (1.9)
Government hospital	102 (98.1)
Drugs used for treatment	n = 104
LAmB (5 mg/Kg single dose) + MF (2.5 mg/Kg/per day for 7 days)	3 (2.8)
LAmB single dose (10 mg/Kg)	77 (74.0)
LAmB multiple doses (5 mg/Kg per day for 3 days)	15 (14.4)
Miltefosine (100mg per day for 28 days)	6 (5.7)
PMIM (11mg/Kg for 10 days) + LAmB (5 mg/Kg single dose)	1 (0.9)
Do not know	1 (0.9)
Did not get treatment because of pregnancy	1 (0.9)
Doctor call for follow-up	n = 104
Yes	63 (60.6)
No	41 (39.4)
Patient went for follow-up	n = 104
Yes	39 (37.5)
No	65 (62.5)
Monitoring during follow-up by the doctor	n = 39
Asked the health status	35 (89.7)
Monitored fever	28 (26.9)
Measured the spleen size	25 (64.1)
Measured the weight	15 (38.4)
Monitored the haemoglobin	22 (56.4)
Observed the skin lesions	3 (7.7)

**Fig. 3:**
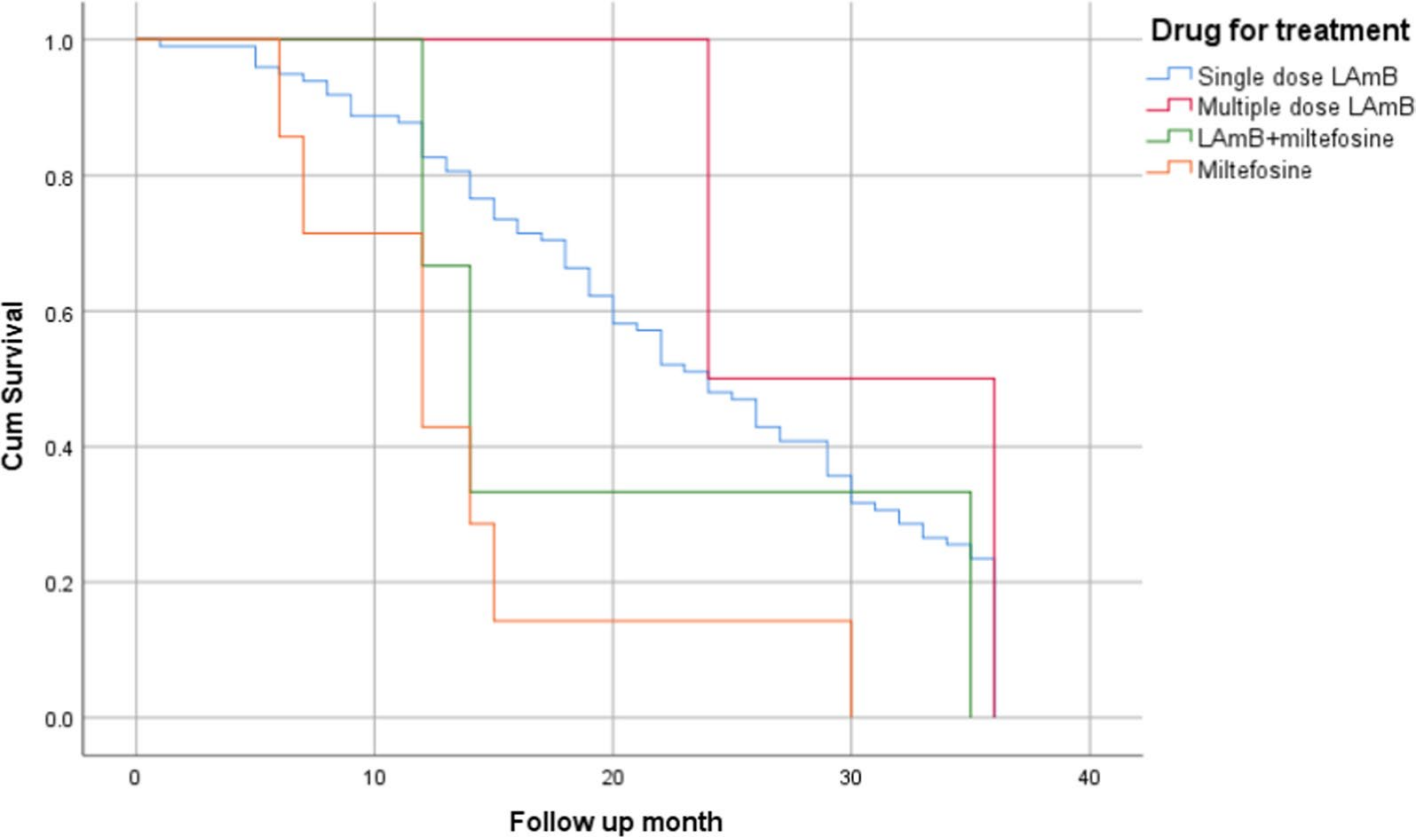
Kaplan Meier curve of duration of follow up of VL treated cases with different drugs

Among the 112 VL treated cases followed up by the clinicians during 1 year period, 24 (21.4%) were followed up only retrospectively and 88 (78.6%) were followed up both retrospectively and prospectively. Among them, 106 (94.6%) were followed in the first follow-up, 80 (86.9%; 76 two follow ups and 4 one follow up) in the second follow-up (at 6 months of first follow-up) and 82 (73.2%; 76 three follow ups, 5 two follow ups and 1 with only one follow up) in the third follow-up (at 12 months of the first follow-up) (Fig. 3). At first follow-up, 4 had fever for more than 2 weeks, 3 had no appetite, 4 had perceived weight loss, and 3 had splenomegaly. Similarly, at second follow-up, 1 had fever, 3 had loss of appetite, 3 had perceived loss of weight, and 2 had splenomegaly. At third follow-up, no one had fever, 1 had loss of appetite, 1 had perceived weight loss and 2 had splenomegaly. However, none of them were VL positive during all follow-ups. However, in between the follow ups, 8 cases reported that they had a VL relapse and got treatment in the hospital. PKDL like skin lesions were found among 8 VL treated cases and among them 2 were PKDL positive. The median weight and mean haemoglobin of the VL treated cases was found increasing at third follow-up as compared to first and second follow ups (Table 3).

**Table 3:** Information on patient follow-up

Indicators	Enrollment % (n)	Follow-up at 6 months % (n)	Follow-up at 12 months % (n)
Total no. of participants (N)	106	80	82
Symptoms of VL			
Fever > 2 weeks	4 (3.8)	1 (1.3)	0
Loss of appetite	3 (2.8)	3 (3.8)	1 (1.2)
Perceived loss of weight	4 (3.8)	3 (3.8)	1 (1.2)
Abdominal enlargement/Splenomegaly	3 (2.8)	2 (2.5)	2 (2.4)
No. of suspected VL patient	0	0	0
No. of reported relapse case of VL within the study period	5 (4.7)	1	2
Symptoms of PKDL			
Lesion on the face, neck, upper and lower limbs	8 (7.5)	0	0
Papules/nodules of skin on the face, neck, upper and lower limbs	0	0	0
No. of PKDL patient	1	1	0
Weight (in Kg) (Median, Min.-Max.)	27.5 (7–115)	27.2 (7–119)	30 (10–117)
Hemoglobin (mg/dl) (mean ± SD)	12.3 ± 2.5	11.6 ± 1.9	12.8 ± 1.8

**Fig. 4:**
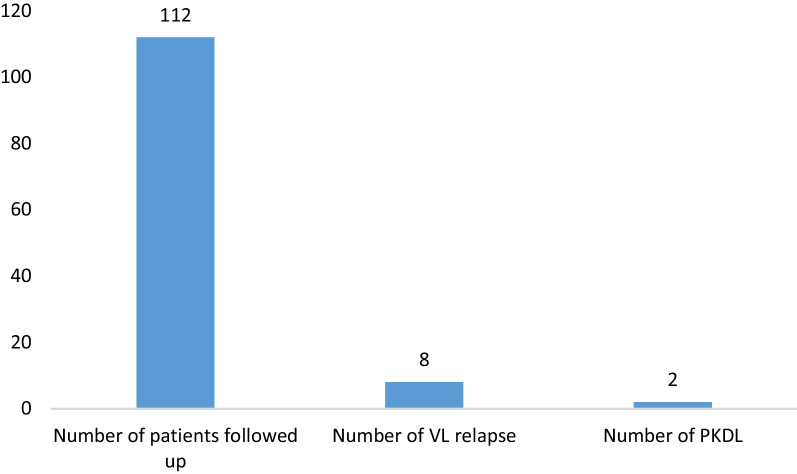
Relapse and PKDL among followed up treated VL cases

It was found that among the total 112 VL treated cases followed up, 8 (7.14%) had relapse and 2 (1.8%) had PKDL (Fig. 4).

Among the relapse cases, 4 (50%) were children less than 10 years of age, and 4 were young adults. The gender distribution was 50% male and 50% female. The drug for treatment was single dose liposomal amphotericin B (10 mg/Kg) in all relapsed cases. The drug for treatment of VL was single dose liposomal amphotericin B (10mg/Kg) in one PKDL case and multiple dose liposomal amphotericin B (5 mg/Kg a day for 3 days) in other. Both PKDL cases were found young female adults.

### The impact of COVID-19 on VL case detection, treatment, reporting, vector control operation and logistic supply chain management

Among 112 VL treated cases followed up during the study period, 66 mentioned that they were diagnosed with VL during the COVID-19 pandemic and lock downs. We interviewed these 66 cases regarding the impact of COVID-19. During this period, among 66 cases, 61 (92.4%) were diagnosed in the government hospitals and 5 (7.6%) were diagnosed in the private hospitals. Among 66 cases, 55 (83.3%) were treated with liposomal amphotericin B, 8 (12.1%) with miltefosine, 2 with combination drugs and 1 with amphotericin B. Fifteen (22.7%) cases went to hospital only 4 weeks after onset of symptoms but 32 (48.5%) went to hospital after 1 week. The majority of the cases (32, 48.5%) were diagnosed with VL within 1 week; however, 10 (15.1%) were diagnosed with VL after 4 weeks or more time. Among 66 cases, 65 (98.5%) started treatment within a week after VL diagnosis. Seven (10.6%) cases had to cancel their appointment during the COVID-19 period due to lack of money (3, 42.8%), fear of COVID-19 (3, 42.8%), unavailability of transportation (1, 14.3%) and doctor not giving an appointment (1, 14.3%). Seventeen cases of VL (25.7%) faced a shortage of diagnostics and drugs for treatment during the COVID-19 lock down period (Table 4).

**Table 4:** Impact of COVID-19 on VL cases

Characteristics	Numbers (n = 66) (%)
Place of diagnosis	
Private hospitals	5 (7.6)
Government hospitals	61 (92.4)
Drugs used for treatment	
Amphotericin_B	1 (1.5)
Liposomal_amphotericin B	55 (83.3)
Miltefosine	8 (12.1)
Combination therapy	2 (3.1)
Time taken to fall ill and seek treatment	
1 week	32 (48.5)
2 weeks	14 (21.2)
3 weeks	5 (7.6)
4 weeks and more	15 (22.7)
Time taken for diagnosis	
1 week	45 (68.2)
2 weeks	7 (10.6)
3 weeks	4 (6.1)
4 weeks and more	10 (15.1)
Time taken to start treatment	
1 week	65 (98.5)
2 weeks	1 (1.5)
Appointment cancelled due to COVID-19	
Yes	7 (10.6)
No	59 (89.4)
Reasons for cancellation (multiple responses)	
Lack of money	3 (42.8)
Fear of COVID-19	3 (42.8)
Unavailability of transportation	1 (14.3)
Doctors did not give appointment	1 (14.3)
Shortage of drugs and treatment	
Yes	17 (25.7)
No	49 (74.2)

According to the VL focal persons, COVID-19 had a huge impact on public health services including VL elimination activities. Active search for VL was not conducted during the COVID-19 period because of a lack of budget, and unavailability of rK39 rapid tests in the district health office. Insecticide spraying was not done during the lock down, and VL drugs were available but in low amounts during the COVID-19 pandemic.

## Discussion

According to Nepal’s National VL Elimination Strategy 2019, the recommended first line drug to treat VL is single dose liposomal amphotericin B [10]. We observed that most of the health workers followed the practice. In few cases with relapse, clinicians used multiple dose liposomal amphotericin B and miltefosine. Such patients were treated only in government hospitals. Monotherapy and combination therapies are completed with shorter duration of hospitalization and have thus been found feasible and acceptable to both clinicians and VL patients [11].

Compared to the number of VL cases reported in recent years, the follow-up status was poor in Okhaldhunga and Kalikot districts which had reported a large number of VL cases in the last year. In other districts, varying but satisfactory numbers of treated VL cases were followed up considering the reported number of VL cases. The national VL treatment protocol recommends follow-up of treated VL cases at one and 6 month of treatment [10]. However, very few clinicians and health workers follow this schedule. Treated VL cases who felt healthy did not understand the need for follow-up. Further, VL is associated with poverty [12, 13] and patients are often unable to cover the transportation costs to go for follow-up. In the hilly districts which now have a large number of VL cases, long distances and difficult terrain pose another barrier for follow-up, particularly for children. Nevertheless, antileishmanial drugs may have side effects and adverse drug reactions after completing the treatment which can affect the quality of life of the treated cases [14]. Therefore, it is very important to follow-up the cases of VL after treatment.

During follow-up, health workers used to monitor fever, spleen size, weight and haemoglobin. Very few health workers monitored skin lesions. PKDL can occur as a skin sequel in kala-azar cases treated with antileishmanial drugs [15]. It was found that not all health workers follow the standard procedure for follow-up. Therefore, clinicians and health workers should be trained on the standard operational procedures for follow-up of VL cases.

It is important to note that the majority of VL treated cases were children less than 10 years of age, many of them students or pre-school children; the others were labourers and farmers. Large number of child cases suggests that there could be indigenous transmission of VL in these districts although some of the districts are in the mountains and hilly regions. The age variation of VL cases may be due to the level of endemicity, peoples’ mobility and other factors [16, 17].

Among the 112 VL treated cases followed up by the clinicians during the study period, 8 had relapse and 2 were PKDL positive. The drug for treatment was single dose liposomal amphotericin B (in all relapsed cases, whereas the drug for treatment of VL was single dose liposomal amphotericin B in one PKDL case and multiple dose liposomal amphotericin B in other PKDL case. There was no case of PKDL in the small fraction of VL treated cases with miltefosine (5.7%) and, with liposomal amphotericin B and miltefosine (2.8%). A previously published study showed that patients had lower relapse but higher PKDL incidence when treated with miltefosine plus paromomycin as compared to those treated with a single dose of liposomal amphotericin B or liposomal amphotericin B plus miltefosine [18]. There is a significant relationship between the rate of development of PKDL and relapse and the treatment regimens for VL [5, 6]. Follow-up studies of up to 36 months in India and 48 months in Bangladesh suggested that most relapses occur during the first year after treatment and that therefore the follow-up period should be at least 12-months.

A study from Brazil mentioned the predictors of relapse to be low haemoglobin, low platelet count before treatment, HIV co-infection, and pneumonia during treatment of VL [19]. In case of miltefosine, young age and male gender are associated with increased risk of VL relapse [20], and the various dosage schedules utilized in miltefosine therapy [21].

In our study, the median weight and mean haemoglobin value was found to increase at third follow-up as compared to first and second follow ups. Weight and haemoglobin are simple markers for treatment success or failures of VL [22] and can also be monitored in peripheral health facilities. If there is no increment in weight and haemoglobin during follow-up of treated cases, the cases may need to be referred to tertiary care centers.

During the COVID-19 lock down period there was not much impact on VL care including case diagnosis and treatment at the hospital. Only few of the cases were diagnosed with VL after a lag time of 4 weeks or more. Among 66 cases, only 7 (10.6%) missed their appointment during the COVID-19 period for lack of money, fear of COVID-19, unavailability of transportation and because the doctor did not give an appointment. The implementation of public health measures to contain the spread of COVID-19 had however a substantial impact on regular vector surveillance and control efforts globally [23]. Nepal, as in other resource-poor countries, faced unparalleled challenges within its healthcare system due to the COVID-19 pandemic, resulting in significant disruptions of crucial healthcare services [24]. About one fourth of VL treated cases faced shortage of diagnostics and drugs during the COVID-19 lock down period. Public health programmes were badly impacted. There was no active search for VL because of lack of budget. Further, rK39 was not available in the district health office during the COVID-19 period to conduct active case detection activities. Insecticide spraying was not done during the lock down. Medicine for VL was supplied in low amounts during COVID-19. In our study, the missed cases during the pandemics could not be analysed. To note is also report of interruption of programmatic VL elimination activities including of indoor residual spraying of insecticides, active case detection and, diagnosis and treatment of VL in the countries of the Indian sub-continent during the COVID-19 period [25].

Treated VL patients lack awareness of the need for follow up and do not seek care unless they feel sick again. Difficult topography is an additional problem for follow-up in hilly districts. Alternative solutions can be applied to address the challenges. The VL focal person of the District Health Office can reach treated cases for follow-up through telephone. Local health workers can be trained and deployed to conduct follow-up.

## Conclusion

Relapse and PKDL among treated VL cases is a challenge for VL elimination and a matter of concern. The national VL elimination programme should be proactive in the follow-up of treated VL cases as per strategy to identify relapse and PKDL and to initiate the treatment at the earliest. This can be done through building capacity of the hospitals and health facilities, and training clinicians and district VL focal persons or health workers from local health facilities to apply the national guidelines including parasitological diagnosis for relapse and skin snip sampling of suspect skin lesions to confirm PKDL, avoiding referral of patients to distant tertiary care hospitals. Although there was no major impact of COVID-19 on VL diagnosis and treatment, public health programmes including active case detection and insecticide spraying for vector control were badly impacted during the COVID-19 lock down.

## Data Availability

All data generated or analysed during this study are included in this published article.
